# Proteolysis at a Specific Extracellular Residue Implicates Integral Membrane CLAG3 in Malaria Parasite Nutrient Channels

**DOI:** 10.1371/journal.pone.0093759

**Published:** 2014-04-03

**Authors:** Wang Nguitragool, Kempaiah Rayavara, Sanjay A. Desai

**Affiliations:** The Laboratory of Malaria and Vector Research, National Institute of Allergy and Infectious Diseases, National Institutes of Health, Rockville, Maryland, United States of America; Université Pierre et Marie Curie, France

## Abstract

The plasmodial surface anion channel mediates uptake of nutrients and other solutes into erythrocytes infected with malaria parasites. The *clag3* genes of *P. falciparum* determine this channel’s activity in human malaria, but how the encoded proteins contribute to transport is unknown. Here, we used proteases to examine the channel’s composition and function. While proteases with distinct specificities all cleaved within an extracellular domain of CLAG3, they produced differing degrees of transport inhibition. Chymotrypsin-induced inhibition depended on parasite genotype, with channels induced by the HB3 parasite affected to a greater extent than those of the Dd2 clone. Inheritance of functional proteolysis in the HB3×Dd2 genetic cross, DNA transfection, and gene silencing experiments all pointed to the *clag3* genes, providing independent evidence for a role of these genes. Protease protection assays with a Dd2-specific inhibitor and site-directed mutagenesis revealed that a variant L1115F residue on a CLAG3 extracellular loop contributes to inhibitor binding and accounts for differences in functional proteolysis. These findings indicate that surface-exposed CLAG3 is the relevant pool of this protein for channel function. They also suggest structural models for how exposed CLAG3 domains contribute to pore formation and parasite nutrient uptake.

## Introduction

Malaria parasites develop and replicate within erythrocytes, contributing to disease severity and pathogen success [1]. This intracellular development requires nutrients from both erythrocyte stores and host plasma. Parasite digestion of hemoglobin provides many needed amino acids [2]–[4], but some nutrients are not available in sufficient quantities in erythrocyte cytosol and must be obtained from external sources [5], [6]. Plasma is the main source of purines, some vitamins, and isoleucine, an essential amino acid absent from human hemoglobin [7]–[10]. The host membrane permeability of these and other nutrients is increased after infection to meet the parasite’s demands [11]–[14].

Patch-clamp studies have implicated the plasmodial surface anion channel (PSAC) in increased uptake by human erythrocytes infected with *P. falciparum* [15]. *P. knowlesi*, a phylogenetically distant malaria parasite, induces remarkably similar channels on rhesus monkey red blood cells [16], but parasites from other genera that invade erythrocytes do not activate similar channels [17]. Interestingly, genetically distinct *P. falciparum* clones yield channels with modest, but reproducible differences in gating or pharmacology, even when erythrocytes from a single donor are used [18], [19]; this suggests a role for parasite-encoded proteins that vary from one clone to another. Because malaria parasites are known to export other proteins having variant sequences to the host membrane [20]–[22], it is possible that parasite proteins mediate increased erythrocyte permeability. Recently, a candidate parasite protein was identified through genetic mapping with ISPA-28, a compound that inhibits PSAC activity only on erythrocytes infected with a specific parasite clone known as Dd2 [23]. The protein, encoded by either *clag3.1* or *clag3.2* in the parasite genome, is trafficked to the host membrane. Consistent with functional studies, this protein is conserved in all malaria parasites and absent from other organisms [24], [25].

How CLAG3 functions to alter erythrocyte permeability is unknown. This 160 kDa protein has no homology to known ion channels and lacks conventional transmembrane domains for formation of stable aqueous pores. The protein belongs to a small conserved family restricted to malaria parasites; some members of this family have been implicated in erythrocyte invasion or cytoadherence [26], [27]. CLAG3 is synthesized late in the intracellular parasite cycle and packaged into rhoptries, specialized organelles within daughter merozoites [28]; there, it associates with two unrelated parasite proteins to form the RhopH complex [29]. Upon merozoite egress from the host cell, this complex is released into the extracellular medium and also found in the erythrocyte compartment of newly invaded cells [30], [31]. Biochemical studies have revealed two populations of CLAG3 protein within infected cells: one that can be stripped from membranes by alkaline treatment and another that is resistant to alkaline extraction but susceptible to cleavage by extracellular proteases [23]. These pools reflect peripheral and integral membrane CLAG3, respectively. Susceptibility to extracellular proteases indicates that at least some of the integral membrane pool localizes to the host erythrocyte membrane. The presence of multiple CLAG proteins, localization to diverse sites in the infected erythrocyte, and distinct subpopulations in protein biochemical studies have all prevented clear insights into how these proteins contribute to host cell permeability. As a transmembrane protein at the erythrocyte surface, CLAG3 may either contribute to the channel pore directly or it may influence PSAC activity *in trans*. As a peripheral membrane protein in host cytosol, it could modulate channel activity via transient interactions with the pore or, more indirectly, through soluble second messengers [14], [32].

Here, we have examined these uncertainties with externally applied proteases. We found that multiple proteases cleave the CLAG3 protein within an extracellular variable loop, but there are significant differences in their effects on transport. Our studies establish a causal link between proteolysis within this loop and reduced PSAC-mediated transport. Site-directed mutagenesis of CLAG3 identified a single variant residue as the responsible protease cleavage site. These studies indicate an involvement of integral membrane CLAG3 at the host membrane and contribute to an evolving structural model for parasite-induced nutrient permeation.

## Materials and Methods

### Protease Treatment and Transport Measurements

*P. falciparum* clones were cultivated in human erythrocytes (Interstate Blood Bank, Memphis, TN) and maintained under 5% O_2_, 5% CO_2_, 90% N_2_ at 37°C. Trophozoite-infected cells were enriched by percoll-sorbitol density gradient centrifugation, washed and resuspended at 5% hematocrit and 25 uL volume in 150 mM NaCl, 20 mM Na_2_HPO_4_, 0.6 mM CaCl_2_, 1 mM MgCl_2_, pH 7.4. Protease treatment was initiated by adding an equal volume of the buffer with freshly dissolved protease at 2 mg/mL (chymotrypsin or trypsin) or 1 mg/mL (pronase E). In each experiment, a no-protease control was identically diluted in protease-free buffer. After a 1 h incubation at 37°C, 2 mM phenylmethylsulfonyl fluoride (PMSF) was added to inhibit serine proteases.

These cells were immediately used to quantify PSAC activity with osmotic lysis as described [13]. Solute uptake was initiated by addition of 950 uL of 280 mM sorbitol, 20 mM HEPES, pH 7.4. Transmittance of 700 nm light transmittance through the cell suspension was used to track the kinetics of osmotic lysis, which results from net uptake of sorbitol via PSAC and subsequent swelling due to water uptake via aquaporins [33]. Protease-mediated inhibition was calculated according to % inhibition = 100* (τ_p_ – τ_o_)/τ_p_, where τ_p_ and τ_o_ are the interpolated time to 50% hemolysis for protease-treated and matched untreated cells, respectively. Estimates of transport inhibition using this approach match those obtained with tracer flux and patch-clamp methods [18], [34], [35]. Solute permeability coefficients and altered selectivity in PSAC mutants have also been quantified with continuous tracking of osmotic lysis kinetics [36], [37].

Protease protection assays examined whether ISPA-28 binding to CLAG3 prevents chymotrypsin-induced inhibition. These experiments were performed as above with the addition of 2.5 μM ISPA-28 during chymotrypsin exposure. Chymotrypsin treatments were increased to 2 h for Dd2-infected cells to yield detectable transport inhibition. After addition of PMSF, infected cells were washed to remove PSAC inhibitor before initiating transport.

### Immunoblots

Immunoblots were performed with a specific anti-CLAG3 antibody raised against a recombinant C-terminal fragment as described previously [23]. Protein samples were denatured, separated by SDS-PAGE, and transferred to nitrocellulose membrane. After blocking (3% fat-free milk in 150 mM NaCl, 20 mM TrisHCl, pH 7.4 with 0.1% Tween20), the antibody was applied at 1∶3000 dilution in blocking buffer. After washing, binding was detected with HRP-conjugated secondary antibodies (Pierce, Rockford, IL) at 1∶3000 dilution and chemiluminescent substrate.

### Linkage Analysis

QTL analysis to identify genomic loci responsible for differences in chymotrypsin sensitivity was performed as described previously [38]. Mean chymotrypsin inhibition values were determined from up to 6 trials for each progeny clone; statistical correlations between this phenotype data and genotypes were sought with R/qtl software (available at http://www.rqtl.org/) as described [39]. A *P = *0.05 significance threshold was determined by permutation analysis. A secondary scan to search for additional QTL was carried out by controlling for the primary chromosome 3 locus as described in the R/qtl software package.

### DNA Transfection

The pHD22Y-120w transfection plasmid, generated previously [23], was subjected to site-directed mutagenesis by whole-plasmid PCR with complementary primers: 5′-TTAGTTGGACACACGCTTATACAACTGGACAACATT-3′ and 5′-AATGTTGTCCAGTTGTATAAGCGTGTGTCCAACTAA-3′ to introduce the I1105Y mutation; 5′-CAACATTTAATTCCCCAATTTACAGATCCTGAATACG-3′ and 5′-CGTATTCAGGATCTGTAAATTGGGGAATTAAATGTTG-3′ for the L1115F mutation. After DpnI digestion of template plasmid, the PCR product was used to transform chemically competent XL-10 gold *E. coli*. After DNA sequencing to confirm the desired mutation, digestion with NotI and PstI released the *clag3.1* gene fragment. The fragment was transferred to fresh pHD22Y backbone and used for allelic exchange transfection of Dd2 parasites. The transfected culture was cycled on and off 5 nM WR99210 every three to four weeks for 4 months prior to PCR detection of homologous recombination of the L1115F mutant plasmid. All experiments were performed with a limiting dilution clone [40].

### Southern Blot

Genomic DNA was digested with BamHI or HindIII, resolved on a 0.7% agarose gel, and transferred to nylon membrane. A digoxigenin-dUTP labeled DNA probe complementary to the *hdhfr* gene on the plasmid backbone was prepared, hybridized to the blot, and washed as described previously [23]. Binding was then detected with anti-digoxigenin-AP Fab fragments at a dilution of 1∶10,000 and CDP-Star substrate (Roche, Indianapolis, IN).

### RT-PCR

Two-step RT-PCR was used to quantify relative expression of the two *clag3* genes in each parasite. RNA was harvested from schizont-stage cultures with TRIzol reagent (Invitrogen, Carlsbad, CA), treated with DNase to remove residual genomic DNA contaminant, and used for reverse transcription (SuperScriptIII and oligo-dT priming, Invitrogen, Carlsbad, CA). PCR was then performed with gene-specific primers and identical quantities of cDNA template in all reactions [23]. Control reactions without reverse transcriptase were used to exclude genomic DNA contamination.

## Results

### Differing Effects of Proteases on CLAG3 Hydrolysis and PSAC-mediated Transport

**Figure 1:**
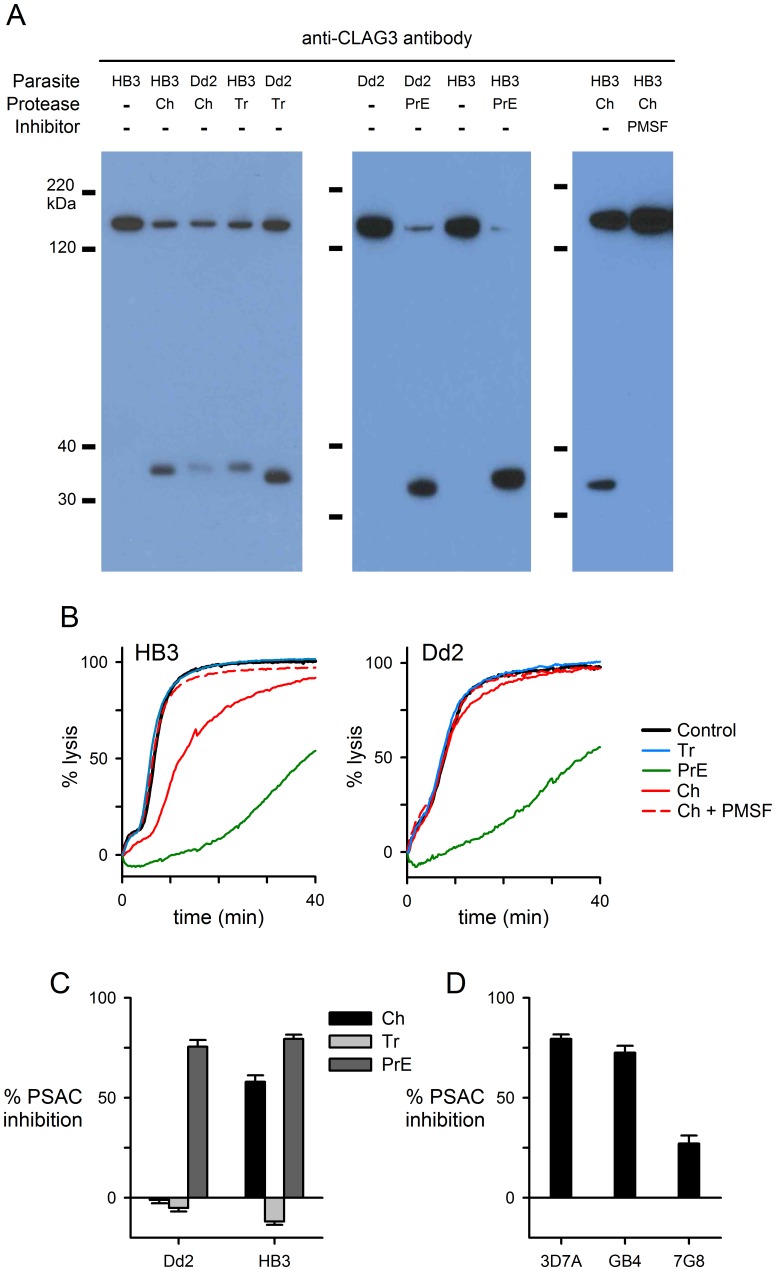
Effects of proteases on CLAG3 and PSAC-mediated transport. (A) Immunoblots showing CLAG3 hydrolysis in HB3 and Dd2 parasites by chymotrypsin (“Ch”), trypsin (“Tr”), or pronase E (“PrE”); mouse anti-CLAG3 generated using a recombinant C-terminal fragment [23]. The band at ∼160 kDa reflects uncleaved CLAG3; a C-terminal proteolysis fragment at ∼35 kDa is visible upon protease treatment. Addition of 2 mM PMSF abolishes cleavage by chymotrypsin. (B) Osmotic lysis kinetics for HB3- and Dd2-infected cells in sorbitol. Control traces represent matched samples not exposed to proteases or PMSF (black traces). While pronase E retards PSAC-mediated osmotic lysis and trypsin is without effect in both parasites (green and blue traces, respectively), chymotrypsin inhibits transport in HB3- but not Dd2-infected cells (red solid traces). (C) Mean ± S.E.M. PSAC inhibition determined from osmotic lysis experiments, normalized to 0% for no protease controls. (D) Mean ± S.E.M. inhibition resulting from chymotrypsin treatment of erythrocytes infected with indicated parasites.

Treatment of infected cells with chymotrypsin or pronase E reduces PSAC-mediated solute transport, but trypsin has negligible effect [23], [41]. We investigated the effects of these proteases on CLAG3 using intact infected erythrocytes and conditions that avoid protein cleavage at intracellular sites [23]. To explore possible effects of polymorphisms, we utilized the Dd2 and HB3 parasite clones, derived originally from patients in Indochina and Honduras respectively. After extracellular protease treatment, we performed denaturing gel electrophoresis and immunoblotting with an antibody that recognizes the CLAG3 C-terminus. With chymotrypsin, trypsin, or pronase E treatment, a single proteolytic fragment of approximately 35 kDa size was detected; 2 mM phenylmethanesulfonylfluoride (PMSF, a serine protease inhibitor) inhibited release of this fragment when added during chymotrypsin treatment (Fig. 1A). With each protease, semi-quantitative comparison to the retained 160 kDa full length CLAG3 indicated that a significant fraction of parasite CLAG3 is exposed and cleaved at the host cell membrane.

Because our antibody was raised against a recombinant C-terminal fragment, the additional band’s size corresponds to the distance of the site of proteolysis from the protein’s C-terminus. Then, the observed ∼ 35 kDa distance puts the cleavage site within or near a 15–30 residue domain that varies amongst sequenced *P. falciparum* clones [42]; the remainder of the protein is highly conserved. Cleavage here is consistent with evidence that this domain is exposed at the host cell surface [23]. Interestingly, the exact size of the cleavage product depended on both protease and parasite genotype. These modest differences in size suggest position-specific cleavage within an exposed variant loop on the polypeptide. This prediction is consistent with the different specificities of these proteases: while chymotrypsin preferentially cleaves peptide amide bonds at aromatic residues, trypsin acts mainly at lysine and arginine residues. Pronase E is a mixture of proteases having broad substrate specificity [43].

We next examined the effects of protease treatment on channel-mediated transport with kinetic measurements of osmotic lysis in sorbitol, a sugar alcohol with high PSAC permeability [13]. In contrast to the qualitatively similar effects of these proteases on CLAG3 hydrolysis, we found significant differences in their effects on transport. In studies using HB3-infected cells, chymotrypsin reduced sorbitol uptake, but trypsin did not inhibit transport (Fig. 1B). Reduced uptake resulted directly from proteolysis because transport inhibition was reversed by addition of 2 mM PMSF, as also seen in immunoblotting of CLAG3 proteolysis (Fig. 1A). We quantified PSAC inhibition by each of the proteases and found marked differences in activity against sorbitol uptake (Fig. 1C). Consistent with nonspecific action against multiple residues, pronase E had the greatest effect on transport into cells infected with either clone. Although modest when compared to the effects of potent PSAC inhibitors [13], the substrate specificities of these proteases and their differential effects on transport provide a molecular handle for examining the channel’s composition and structure.

Trypsin’s inability to reduce transport is in sharp contrast to its proteolytic action against CLAG3. This, along with the large number of host and parasite proteins on the erythrocyte surface, suggests that CLAG3 hydrolysis might not be directly responsible for reduced transport after treatment with any of these proteases. One possibility is that other proteins define the PSAC pore; selective cleavage of these other unknown channel components by chymotrypsin or pronase E, but not by trypsin may then account for the lack of trypsin effect on transport. Another possibility is that proteolysis of CLAG3 is fully responsible for transport inhibition, but that the extent of inhibition depends on the specific cleavage site(s) on the polypeptide.

Surprisingly, chymotrypsin’s effect on transport depended on parasite genotype, with a significantly greater channel block seen in studies using HB3 than Dd2 parasites (Fig. 1B and 1C; *P*<10^−9^, Student’s *t* test, determined from 10 trials each). While this difference appears to parallel a greater efficacy for CLAG3 proteolysis in HB3 (Fig. 1A), the causal link between proteolysis and channel inhibition is uncertain. Three additional clones each exhibited characteristic reductions in transport upon chymotrypsin treatment, suggesting polymorphisms in one or more parasite proteins at the host cell surface (Fig. 1D).

### Linkage Analysis, Allelic Exchange, and Gene Switching Implicate *clag3* Genes in Chymotrypsin Sensitivity

**Figure 2:**
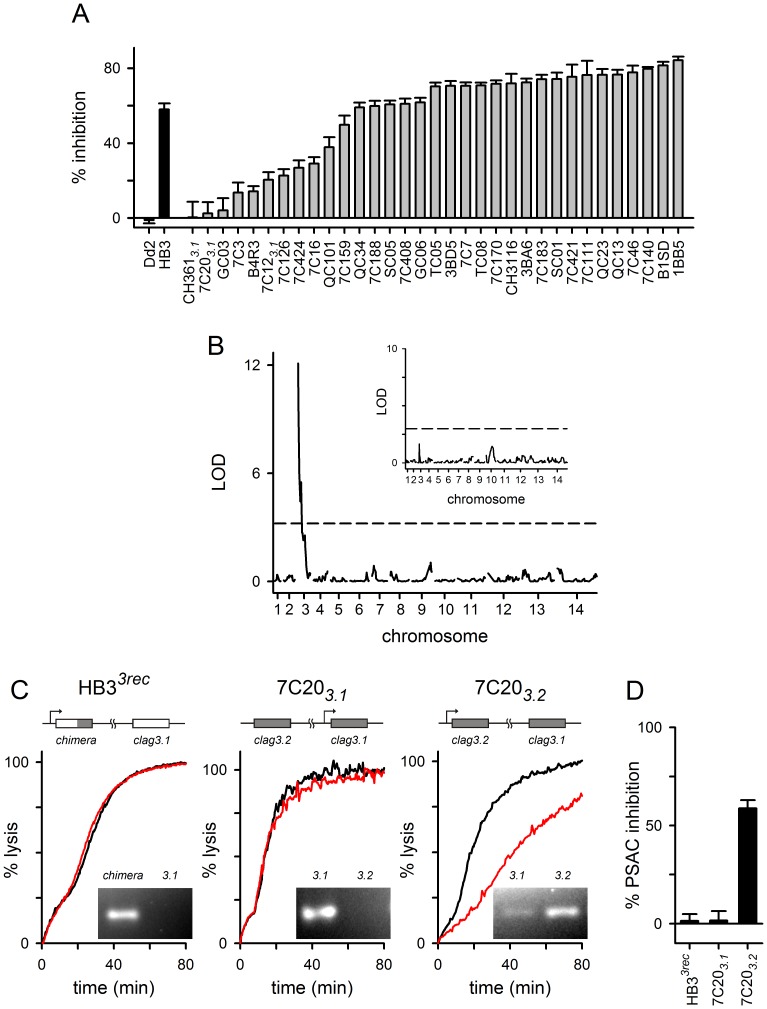
*clag3* genes accounts for the differing sensitivities to chymotrypsin. (A) Mean ± S.E.M. block of sorbitol uptake by chymotrypsin treatment on indicated parental lines and progeny clones (black and gray bars, respectively). (B) Logarithm of odds (LOD) scores from a primary scan of QTL associated with PSAC inhibition. The peak at the 5′ end of chromosome 3 contains the two *clag3* genes. The *P* = 0.05 significance threshold (dashed horizontal line) was calculated from 1000 permutations. Inset shows results from a secondary scan for additional QTL after controlling for the *clag3* locus. No other loci reached the *P* = 0.05 threshold (dashed horizontal line). (C) Osmotic lysis kinetics for indicated parasites after selection for expression of a specific *clag3* gene. Black and red traces represent no protease control and chymotrypsin-treated cells, respectively. The ribbon schematic at the top of each panel shows the gene structure for the two *clag3* genes in each parasite with active transcription indicated by a bent arrow. The *clag3* gene resulting from allelic exchange in HB3*^3rec^* has a gray shaded 3′ end to indicate the fragment derived from Dd2 (“*chimera*”). For each parasite, relative expression of the two paralogs is shown with an ethidium-stained gel at the bottom right of each panel. (D) Mean ± S.E.M. chymotrypsin-induced inhibition for each selected parasite.

We next utilized an available HB3×Dd2 genetic cross to examine inheritance of chymotrypsin effect on transport. Erythrocytes infected with each progeny clone were treated with chymotrypsin under standardized conditions and used for sorbitol uptake measurements (Fig. 2A). To reduce possible contributions from switching between expression of the two *clag3* genes, the 7C20, 7C12, and CH361 progeny clones were examined after selection for *clag3.1* expression because Dd2, from which these daughters inherit both *clag3* genes, is transcription-incompetent for *clag3.2* [23]. Under these conditions, there was negligible transport inhibition upon chymotrypsin treatment (progeny clones labeled 7C20*_3.1_*, 7C12*_3.1_*, and CH361*_3.1_* in Fig. 2A). Because other daughters exhibited a range of chymotrypsin sensitivities, we used QTL analysis to search for contributing genomic loci. A primary scan identified a single significant locus at the 5′ end of chromosome 3 (LOD score of 12.1, Fig. 2B), which includes both *clag3* genes and 40 additional genes. A secondary scan, performed after controlling for this locus, did not find additional loci reaching a *P = *0.05 significance threshold (Fig. 2B, inset).

Sixteen of the genes in the mapped chromosome 3 locus carry the PEXEL domain, which has been implicated in trafficking of parasite proteins to host cytosol and erythrocyte membranes [44]. This raises the possibility that additional channel determinants may be present in this locus. Such syntenic genome arrangement of genes encoding ion channel subunits is well-established in other organisms [45]. To explore this possibility and to assess the contribution of *clag3* genes, we performed experiments with HB3*^3rec^*, a parasite that carries a chimeric *clag3* gene through allelic exchange transfection [23]. The chimeric gene derives its 5′UTR and initial coding regions from the HB3 *clag3.2* gene, but the 3′ end of the open reading frame encoding the last 418 residues is from the Dd2 *clag3.1* gene. We previously selected an HB3*^3rec^* subpopulation that preferentially expresses this chimeric gene and silences the unmodified HB3 *clag3.1* paralog. Under these conditions, the predominant *clag3* product made by these cells has the variable domain of Dd2 CLAG3.1 exposed at the host cell surface. Although this parasite is otherwise isogenic with HB3, chymotrypsin treatment revealed negligible PSAC inhibition matching that seen with Dd2 parasites (Fig. 2C and 2D). Thus, the *clag3* genes adequately account for heritable differences in PSAC susceptibility to chymotrypsin.

We examined the contribution of CLAG3 further with studies of the 7C20 progeny clone after selection for expression of either *clag3.1* or *clag3.2* [38], referred to as 7C20*_3.1_* and 7C20*_3.2_* respectively. Parasites in these cultures have identical genome sequences, but use epigenetic marks to achieve preferential expression of one or the other *clag3* gene [30], [46], [47]. Whereas erythrocytes infected with 7C20*_3.1_* exhibited unchanged sorbitol transport kinetics upon chymotrypsin treatment, those infected with 7C20*_3.2_* exhibited reduced transport kinetics (red traces, Fig. 2C and 2D). These findings further implicate CLAG3 in transport inhibition due to proteolysis. Because progeny clones and other parasite lines exhibit differing expression ratios for the two *clag3* genes in steady-state cultures [23], [47], [48], these findings also account for the non-parental phenotypes observed in some progeny clones (Fig. 2A). Finally, our experiments suggest that the Dd2 *clag3.1* gene, as inherited and preferentially expressed by 7C20*_3.1_*, carries one or more unique polymorphisms that protect the channel from chymotrypsin’s inhibitory effects; these predicted polymorphisms distinguish Dd2 *clag3.1* from the Dd2 *clag3.2* gene and from either *clag3* gene of unrelated parasites.

### ISPA-28 Binding and PSAC Block Protects Channels from Chymotrypsin-mediated Inhibition

**Figure 3:**
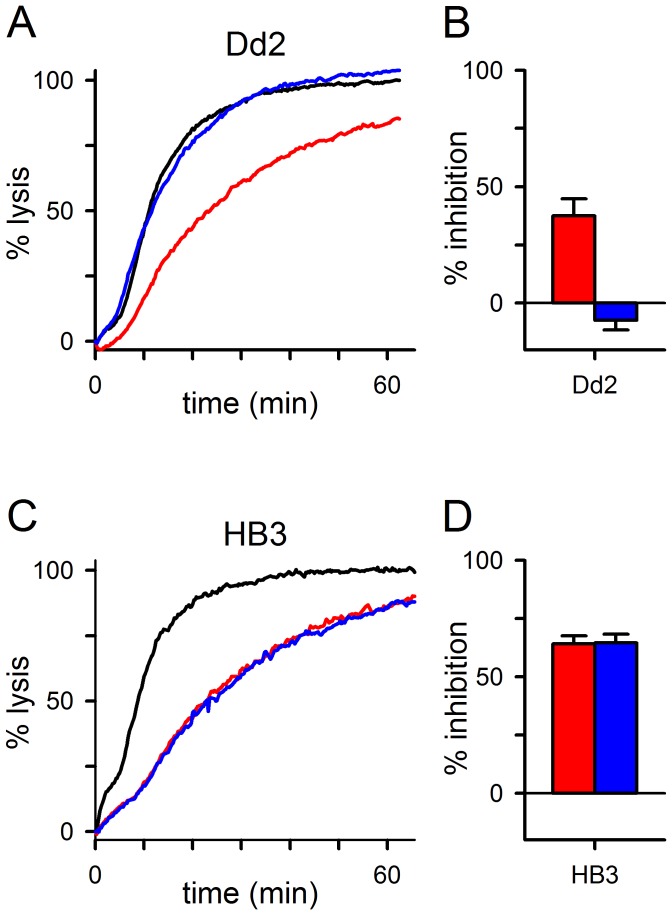
ISPA-28 protects Dd2 channels from proteolytic loss of function. (A) Osmotic lysis kinetics for Dd2-infected cells after a 2 h chymotrypsin treatment in the absence or presence of 2.5 μM ISPA-28 (red and blue traces, respectively); black trace represents a matched control subjected to preincubation without protease or ISPA-28. Notice that addition of ISPA-28 interferes with chymotrypsin action on channels. (B) Mean ± S.E.M. chymotrypsin-induced inhibition without and with ISPA-28 addition (red and blue bars, respectively; *n = *9). (C) Osmotic lysis kinetics for HB3-infected cells due to 1 h chymotrypsin treatment in the absence or presence of 2.5 μM ISPA-28 (red and blue traces, respectively). ISPA-28 does not restore transport to the control level (black trace). (D) Mean ± S.E.M. inhibition without and with ISPA-28 addition (red and blue bars, respectively; *n = *6).

The above experiments with HB3*^3rec^* restrict the functionally relevant polymorphisms to the C-terminal CLAG3 fragment because this transfection fully confers Dd2’s low chymotrypsin susceptibility to HB3 parasites; the small molecule PSAC inhibitor ISPA-28 is also known to block transport through action upon this fragment [23]. To test the role of this CLAG3 fragment in channel-mediated transport, we designed a protease protection assay using ISPA-28. Because ISPA-28 is only effective as an inhibitor of Dd2 channels (*K_0.5_* values of 56 nM and 43 μM for Dd2 and HB3 channels, respectively), it was necessary to identify conditions for proteolytic PSAC inhibition on this parasite. We found that increasing the duration of chymotrypsin treatments to 2 h yielded reproducible inhibition of Dd2 channels (Fig. 3A, red trace). This unusual delay in functional proteolysis may reflect slower cleavage kinetics due to effects of neighboring residues or secondary protein structure [49]. There may also be a lag period if transport inhibition in this parasite requires multiple cleavage events, either within a single CLAG3 polypeptide or on separate unknown channel subunits.

Addition of 2.5 μM ISPA-28 during this longer chymotrypsin treatment followed by washing to remove the inhibitor fully abolished protease-mediated inhibition (Fig. 3A, blue trace; compare to black trace, no protease control; Fig. 3B, *P* *= *10^−4^, paired *t* test for *n = *9 trials). Parallel experiments using HB3 parasites revealed no protection by ISPA-28 (*P = *0.8, *n = *6), consistent with this inhibitor’s inability to bind and block HB3 channels (Fig. 3C and 3D). Importantly, the failure to protect HB3 channels from proteolysis excludes an inhibitory effect of ISPA-28 on chymotrypsin activity. Instead, these findings suggest overlapping external sites on Dd2 channels for action of ISPA-28 and chymotrypsin.

### Site-directed Mutagenesis Implicates a Polymorphic Residue in the Dd2 *clag3.1* Variable Domain

**Figure 4:**
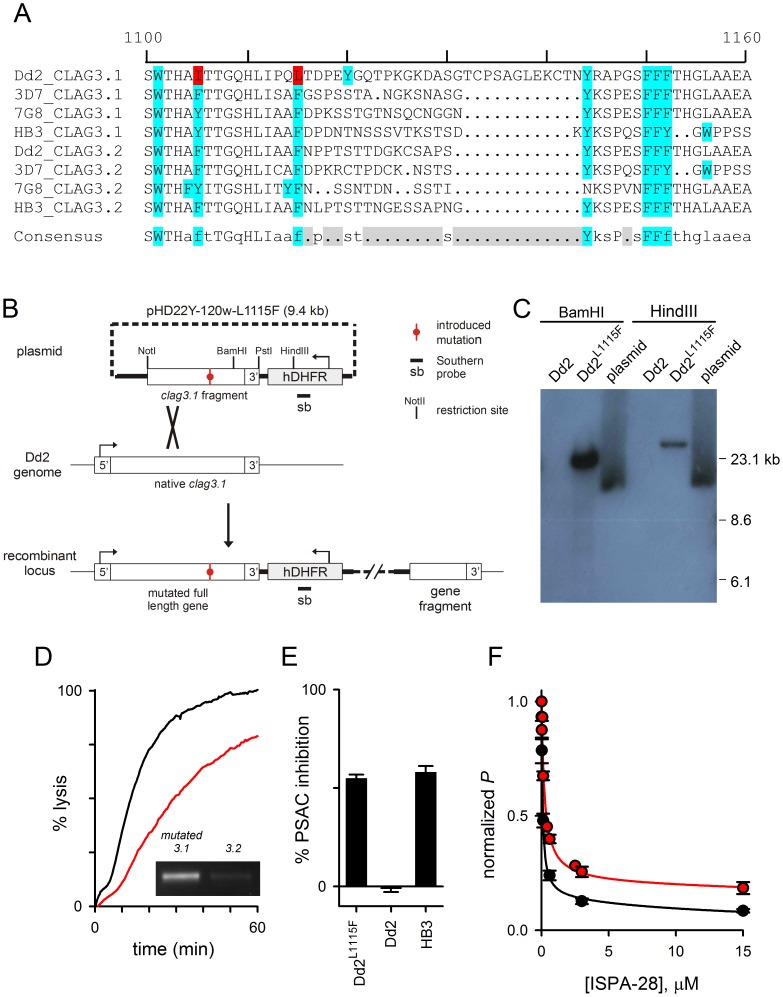
A variant extracellular residue accounts for PSAC resistance to chymotrypsin in Dd2 parasites. (A) Multiple sequence alignment of an extracellular loop on indicated CLAG3.1 and CLAG3.2 sequences, representing geographically divergent parasites (Dd2, from Indochina; 3D7, probably Africa; 7G8 Brazil; HB3, Honduras). A variable segment is apparent in gray shading (Consensus); residues susceptible to chymotrypsin cleavage are shown in blue. Two sites refractory to cleavage in Dd2 CLAG3.1 are highlighted in red. (B) Schematic showing the allelic exchange strategy to introduce a single mutation in the Dd2 *clag3.1* gene. Plasmid carrying the mutation is shown at the top; single homologous recombination into the Dd2 genome produces an intact full-length gene with a single site mutation and unchanged UTR sequences. (C) Southern blot showing integration of plasmid into the Dd2^L1115F^ genome. Indicated DNA samples were digested and probed with an hDHFR-specific probe. Dd2 is not recognized by this probe, but Dd2^L1115F^ yields a single band whose size differs from that of the plasmid. (D) Osmotic lysis kinetics for Dd2^L1115F^ without and with 1h chymotrypsin treatment (black and red traces, respectively). Inset shows preferential expression of the mutated *clag3.1* gene in this clone. (E) Mean ± S.E.M. chymotrypsin-induced inhibition for indicated parasites. (F) Dose responses for inhibition of sorbitol permeability (*P*) by ISPA-28. Black and red symbols represent mean ± S.E.M. inhibition for Dd2 and Dd2^L1115F^ parasites, respectively. Solid lines represent best fits to the sum of two Langmuir isotherms.

We next used computational analysis to identify polymorphic residues on Dd2 CLAG3.1 that may account for resistance to functional proteolysis. We searched for chymotrypsin cleavage sites conserved in the CLAG3.1 and CLAG3.2 sequences of HB3, 3D7A, and 7G8 as well as the CLAG3.2 of Dd2, but changed to protease-insensitive residues in Dd2 CLAG3.1. Based on preferential proteolysis after tyrosine, tryptophan, and phenylalanine resides [50], only two sites within the full-length sequences, I1105 and L1115 (Fig. 4A, red highlighting), met our requirements. Of note, the leucine at residue 1115 in Dd2 CLAG3.1 may be cleaved by chymotrypsin, albeit at reduced rates [50]. Either residue may account for the chymotrypsin insensitivity of Dd2 channels because both are within the C-terminal fragment transferred to HB3*^3rec^* by allelic exchange (Fig. 2C). In the seven other CLAG3 sequences shown in Fig. 4A, these positions are conserved aromatic residues that should be susceptible to proteolysis by chymotrypsin (blue highlighting, Fig. 4A). Both are just upstream of the variable domain (gray shading, consensus).

To test the contributions of these candidate residues, we designed an allelic exchange strategy using a plasmid carrying a Dd2 *clag3.1* gene fragment starting at 1989 bp from the start codon through the end of the open reading frame and including a native 441 bp 3′ UTR sequence (Fig. 4B). We then engineered site-specific mutations in this plasmid by whole-plasmid amplifications with complementary primers carrying desired mutations. Then, transfection of Dd2 cultures followed by single homologous recombination into the parasite genome should produce a full-length *clag3.1* gene encoding a protein with a single desired mutation. No other sites in the Dd2 *clag3.1* gene or flanking untranslated sequences should be modified by this approach.

We prepared separate plasmids to introduce the I1105Y and L1115F mutations and transfected Dd2 parasites. Although the plasmid to introduce I1105Y did not integrate, PCR successfully detected integration of the plasmid for the L1115F mutation. Subsequent limiting dilution cloning yielded the Dd2^L1115F^ clone, which was confirmed to have a single integration event by Southern blotting (Fig. 4C). DNA sequencing revealed homologous integration into Dd2 *clag3.1* at an upstream site to produce a stable mutant with a single desired mutation. RT-PCR demonstrated that this mutant allele is preferentially expressed (Fig. 4D, inset), as also previously demonstrated for untransfected Dd2 parasites [38].

We then examined chymotrypsin effect on channel function in the transfectant. Without chymotrypsin, sorbitol transport was not significantly altered, as indicated by an unchanged halftime for osmotic lysis (Fig. 4D, black trace); this observation suggests that the mutated residue does not play a critical role in channel selectivity or transport rates. Chymotrypsin treatment, however, revealed marked inhibition (Fig. 4D, red trace). This inhibition differed significantly from that seen with untransfected Dd2 parasites but was indistinguishable from that of HB3 (Fig. 4E, *n = *11, *P*<10^−9^ and 0.89 relative to Dd2 and HB3, respectively), suggesting that the polymorphic 1115 residue is a primary determinant of chymotrypsin-induced transport inhibition. We also found that channels produced by Dd2^L1115F^ were less well inhibited by ISPA-28 than those of the parental Dd2 line (Fig. 4F, *P*<0.01 at both 3 and 10 μM concentrations), suggesting that L1115 contributes to ISPA-28 binding. This change in inhibitor efficacy is consistent with the proposal of overlapping sites for proteolysis and ISPA-28 binding, as suggested by protease protection studies (Fig. 3).

Although the L1115F mutation can account quantitatively for the difference in protease susceptibility of Dd2 and HB3 channels, I1105Y, the other polymorphic residue described above, may still contribute to functional proteolysis in chymotrypsin-sensitive parasites. It is unclear why allelic exchange could not be achieved at that site, but it is possible that mutation at that site is incompatible with channel function in the Dd2 *clag3.1* genetic background.

## Discussion

The malaria parasite CLAG3 protein contributes to nutrient channels through unknown mechanisms. Mechanistic understanding has been hindered by the lack of CLAG3 homology to known ion channels, the absence of typical transmembrane domains, and the observation that the protein can be either peripheral or integral to membranes within the infected erythrocyte. Here, we determined that integral membrane CLAG3 in the host membrane represents the relevant protein pool for contribution to channel activity. We found that although chymotrypsin, trypsin, and pronase E all cleave CLAG3 within a surface-exposed variable domain, their effects on channel function are more complex. While trypsin does not block transport, chymotrypsin inhibits PSAC at levels that depend on parasite genotype. Because these proteases act on numerous erythrocyte surface proteins, the molecular basis was unclear [41]. Our linkage analysis, allelic exchange, and gene expression studies indicate that proteolysis of CLAG3 alone can account for transport inhibition by chymotrypsin. Site-directed mutagenesis then implicated the variant L1115F residue lying within an extracellular protein loop.

Both our protease protection assay and the altered inhibitor efficacy in the Dd2^L1115F^ parasite implicate an ISPA-28 binding site defined, at least in part, by the CLAG3 variable domain. This small molecule inhibitor was identified by a high-throughput screen and was instrumental in linkage analysis studies that led to the identification of the *clag3* genes [23]. However, because genetic mapping is an agnostic approach that, in isolation, is unable to reveal mechanistic insights, those studies could not distinguish possible contributions of CLAG3 protein to transport. This uncertainty has been accentuated as some studies have proposed activation of quiescent host channels by parasite enzymes or soluble modulators [51].

**Figure 5:**
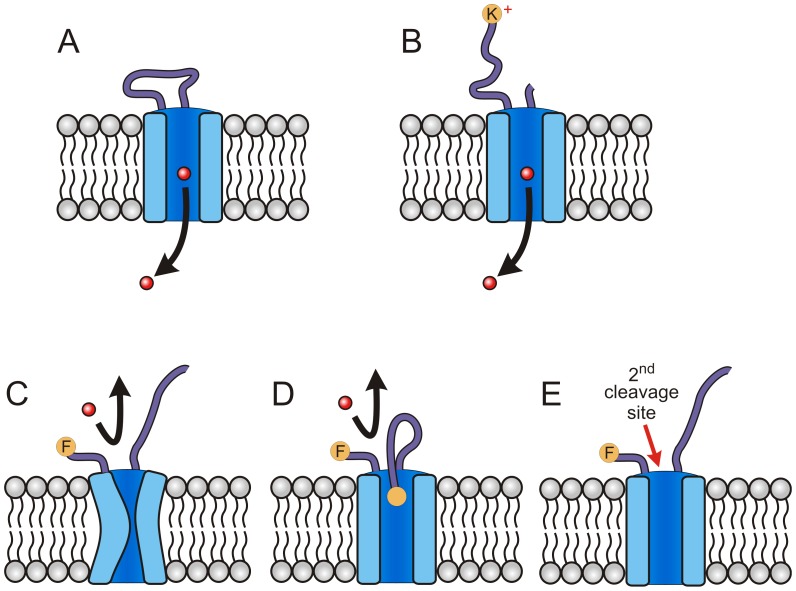
Schematic showing models for protease action on PSAC. (A) Functional channel with a variable extracellular loop and permeating solute (red circle and arrow). (B) Trypsin digestion of extracellular loop, yielding a positively charged end. Solute transport is preserved. (C-E) Possible models of chymotrypsin inhibition in sensitive clones. Panel C shows a collapsed pore due to proteolysis at a critical site in the extracellular loop. Panel D shows steric hindrance of the channel pore, which may prevent solute permeation. Panel E shows cleavage at additional site(s) exposed after cleavage of the extracellular loop.

Our new biochemical studies should help clarify how CLAG3 contributes to transport. Because they demonstrate a role for integral membrane CLAG3, they exclude models with CLAG3 functioning as only a soluble enzymatic activator of channels. The critical L1115F polymorphism influences protease susceptibility and inhibitor efficacy, but does not affect transport rates in the absence of biochemical interventions. When combined with this residue’s localization to a variant extracellular loop, these findings are difficult to reconcile with an active site on an enzyme, and instead suggest that this protein loop is a stable component of the functional channel (Fig. 5A). Sequencing studies have determined that this extracellular protein loop is highly variable amongst parasite clones obtained from divergent locations [52]. Thus, this loop is probably unstructured; it appears to serve a scaffolding role as it holds functional domains that flank it together. Our findings provide experimental evidence as trypsin-mediated cleavage at one or more arginines and lysines within this extracellular loop does not interfere with solute transport (Fig. 4A). Because the trypsin-cleaved polypeptide transports solutes with unchanged rates, an intact extracellular loop at this site is not essential for preserving channel integrity (Fig. 5B). In contrast, CLAG3 sequences flanking this variable domain are conserved in geographically divergent parasite lines; because these flanking sequences are resistant to proteolysis, our biochemical studies suggest either a globular structure that conceals protease-susceptible residues or that the variable domain is bounded on both sides by transmembrane domains.

How then do we account for the effects of pronase E and chymotrypsin on transport? In one model, cleavage at only some sites within this extracellular loop interferes with channel structure, possibly through global conformational changes that collapse the pore or lead to subunit dissociation (Fig. 5C); these changes may be reversible and do not necessarily abolish transport. Such site-dependent effects of proteolysis on stability and function are well-established for channels and other enzymes [53], [54]. Consistent with this model, we saw the greatest loss of transport with pronase E, a promiscuous protease that should act on most critical sites.

In another model, cleavage at certain sites produces free ends that interfere with solute access to the pore through steric hindrance (Fig. 5D). Such a mechanism has been supported for PSAC inhibition by *N*-hydroxysuccinimide esters, where increasing lengths of the modifying agent were associated with greater block durations in single channel recordings [55]. Because dissolved cations are known to be repelled by positive residues that line the pore [55], this model may also account for weak inhibition by trypsin: the positively charged terminal side-chain resulting from cleavage at lysine and arginine residues may be similarly repelled (Fig. 5B), yielding relatively less steric hindrance of the pore.

In a third model, there may be additional proteolytic sites required for loss of PSAC function; these additional sites may be either on CLAG3 or on other proteins that are subunits of the channel; these other proteins are unknown but may be encoded by either the parasite or the host. In this scenario, the L1115F residue implicated by our studies would need to play a permissive role in light of the quantitatively complete change in chymotrypsin susceptibility observed in the Dd2^L1115F^ transfectant. One possibility is that cleavage at this residue renders other sites accessible to protease attack and subsequent loss of channel function (Fig. 5E, 2^nd^ cleavage site).

Precise determination of which, if any, of the above models is correct remains critical for understanding permeation through this unusual channel and uncovering how the parasite remodels its host cell. In addition to the Dd2-specific ISPA-28, inhibitors that interact with conserved sites on the channel have been found through high-throughput screens. Because these compounds kill malaria parasites in culture and have supported an essential role of PSAC in parasite nutrient acquisition [38], this ion channel is being pursued as an antimalarial target. Conservation of *clag* genes in malaria parasites and the greater accessibility of bloodstream antimalarials to this target on the host cell surface should stimulate further interest in drug discovery or vaccine development [56]. Additional studies that map channel topology at the cell surface and identify critical functional domains should help guide antimalarial discovery and development.

## References

[1] Cowman AF, Crabb BS (2006) Invasion of red blood cells by malaria parasites. Cell 124: 755–766. 16497586 10.1016/j.cell.2006.02.006

[2] Rosenthal PJ, McKerrow JH, Aikawa M, Nagasawa H, Leech JH (1988) A malarial cysteine proteinase is necessary for hemoglobin degradation by *Plasmodium falciparum* . J Clin Invest 82: 1560–1566. 3053784 10.1172/JCI113766 PMC442723

[3] Liu J, Gluzman IY, Drew ME, Goldberg DE (2005) The role of *Plasmodium falciparum* food vacuole plasmepsins. J Biol Chem 280: 1432–1437. 15513918 10.1074/jbc.M409740200

[4] Kerr ID, Lee JH, Pandey KC, Harrison A, Sajid M, et al (2009) Structures of falcipain-2 and falcipain-3 bound to small molecule inhibitors: implications for substrate specificity. J Med Chem 52: 852–857. 19128015 10.1021/jm8013663 PMC2651692

[5] Homewood CA, Neame KD (1974) Malaria and the permeability of the host erythrocyte. Nature 252: 718–719. 4612377 10.1038/252718a0

[6] Kutner S, Breuer WV, Ginsburg H, Aley SB, Cabantchik ZI (1985) Characterization of permeation pathways in the plasma membrane of human erythrocytes infected with early stages of *Plasmodium falciparum*: association with parasite development. J Cell Physiol 125: 521–527. 2999164 10.1002/jcp.1041250323

[7] Asahi H, Kanazawa T, Kajihara Y, Takahashi K, Takahashi T (1996) Hypoxanthine: a low molecular weight factor essential for growth of erythrocytic *Plasmodium falciparum* in a serum-free medium. Parasitology 113: 19–23. 8710411 10.1017/s0031182000066233

[8] El Bissati K, Zufferey R, Witola WH, Carter NS, Ullman B, et al (2006) The plasma membrane permease PfNT1 is essential for purine salvage in the human malaria parasite *Plasmodium falciparum* . Proc Natl Acad Sci USA 103: 9286–9291. 16751273 10.1073/pnas.0602590103 PMC1482602

[9] Saliba KJ, Horner HA, Kirk K (1998) Transport and metabolism of the essential vitamin pantothenic acid in human erythrocytes infected with the malaria parasite *Plasmodium falciparum* . J Biol Chem 273: 10190–10195. 9553068 10.1074/jbc.273.17.10190

[10] Istvan ES, Dharia NV, Bopp SE, Gluzman I, Winzeler EA, et al (2011) Validation of isoleucine utilization targets in *Plasmodium falciparum* . Proc Natl Acad Sci USA 108: 1627–1632. 21205898 10.1073/pnas.1011560108 PMC3029723

[11] Ginsburg H, Kutner S, Zangwil M, Cabantchik ZI (1986) Selectivity properties of pores induced in host erythrocyte membrane by *Plasmodium falciparum*. Effect of parasite maturation. Biochim Biophys Acta 861: 194–196. 3530325 10.1016/0005-2736(86)90579-1

[12] Kang M, Lisk G, Hollingworth S, Baylor SM, Desai SA (2005) Malaria parasites are rapidly killed by dantrolene derivatives specific for the plasmodial surface anion channel. Mol Pharmacol 68: 34–40. 15843600 10.1124/mol.104.010553

[13] Pillai AD, Pain M, Solomon T, Bokhari AA, Desai SA (2010) A cell-based high-throughput screen validates the plasmodial surface anion channel as an antimalarial target. Mol Pharmacol 77: 724–733. 20101003 10.1124/mol.109.062711 PMC2872968

[14] Bouyer G, Cueff A, Egee S, Kmiecik J, Maksimova Y, et al (2011) Erythrocyte peripheral type benzodiazepine receptor/voltage-dependent anion channels are upregulated by *Plasmodium falciparum* . Blood 118: 2305–2312. 21795748 10.1182/blood-2011-01-329300

[15] Desai SA (2005) Open and closed states of the plasmodial surface anion channel. Nanomedicine 1: 58–66. 17292059 10.1016/j.nano.2004.11.001

[16] Lisk G, Desai SA (2005) The plasmodial surface anion channel is functionally conserved in divergent malaria parasites. Eukaryot Cell 4: 2153–2159. 16339732 10.1128/EC.4.12.2153-2159.2005 PMC1317498

[17] Alkhalil A, Hill DA, Desai SA (2007) Babesia and plasmodia increase host erythrocyte permeability through distinct mechanisms. Cell Microbiol 9: 851–860. 17087736 10.1111/j.1462-5822.2006.00834.x

[18] Alkhalil A, Cohn JV, Wagner MA, Cabrera JS, Rajapandi T, et al (2004) *Plasmodium falciparum* likely encodes the principal anion channel on infected human erythrocytes. Blood 104: 4279–4286. 15319279 10.1182/blood-2004-05-2047

[19] Alkhalil A, Pillai AD, Bokhari AA, Vaidya AB, Desai SA (2009) Complex inheritance of the plasmodial surface anion channel in a *Plasmodium falciparum* genetic cross. Mol Microbiol 72: 459–469. 19320831 10.1111/j.1365-2958.2009.06661.x PMC2702155

[20] Hviid L (2010) The role of *Plasmodium falciparum* variant surface antigens in protective immunity and vaccine development. Hum Vaccin 6: 84–89. 19823032 10.4161/hv.6.1.9602

[21] Boddey JA, Moritz RL, Simpson RJ, Cowman AF (2009) Role of the Plasmodium export element in trafficking parasite proteins to the infected erythrocyte. Traffic 10: 285–299. 19055692 10.1111/j.1600-0854.2008.00864.x PMC2682620

[22] van Ooij C, Tamez P, Bhattacharjee S, Hiller NL, Harrison T, et al (2008) The malaria secretome: from algorithms to essential function in blood stage infection. PLoS Pathog 4: e1000084. 18551176 10.1371/journal.ppat.1000084 PMC2408878

[23] Nguitragool W, Bokhari AA, Pillai AD, Rayavara K, Sharma P, et al (2011) Malaria parasite *clag3* genes determine channel-mediated nutrient uptake by infected red blood cells. Cell 145: 665–677. 21620134 10.1016/j.cell.2011.05.002 PMC3105333

[24] Kaneko O (2007) Erythrocyte invasion: vocabulary and grammar of the Plasmodium rhoptry. Parasitol Int 56: 255–262. 17596999 10.1016/j.parint.2007.05.003

[25] Crowley VM, Rovira-Graells N, de Pouplana LR, Cortes A (2011) Heterochromatin formation in bistable chromatin domains controls the epigenetic repression of clonally variant *Plasmodium falciparum* genes linked to erythrocyte invasion. Mol Microbiol 80: 391–406. 21306446 10.1111/j.1365-2958.2011.07574.x

[26] Trenholme KR, Gardiner DL, Holt DC, Thomas EA, Cowman AF, et al (2000) *clag9*: A cytoadherence gene in *Plasmodium falciparum* essential for binding of parasitized erythrocytes to CD36. Proc Natl Acad Sci USA 97: 4029–4033. 10737759 10.1073/pnas.040561197 PMC18136

[27] Counihan NA, Kalanon M, Coppel RL, de Koning-Ward TF (2013) Plasmodium rhoptry proteins: why order is important. Trends Parasitol 29: 228–236. 23570755 10.1016/j.pt.2013.03.003

[28] Kaneko O, Yim Lim BY, Iriko H, Ling IT, Otsuki H, et al (2005) Apical expression of three RhopH1/Clag proteins as components of the *Plasmodium falciparum* RhopH complex. Mol Biochem Parasitol 143: 20–28. 15953647 10.1016/j.molbiopara.2005.05.003

[29] Kaneko O, Tsuboi T, Ling IT, Howell S, Shirano M, et al (2001) The high molecular mass rhoptry protein, RhopH1, is encoded by members of the *clag* multigene family in *Plasmodium falciparum* and *Plasmodium yoelii* . Mol Biochem Parasitol 118: 223–231. 11738712 10.1016/s0166-6851(01)00391-7

[30] Cortes A, Carret C, Kaneko O, Yim Lim BY, Ivens A, et al (2007) Epigenetic silencing of *Plasmodium falciparum* genes linked to erythrocyte invasion. PLoS Pathog 3: e107. 17676953 10.1371/journal.ppat.0030107 PMC1937010

[31] Vincensini L, Fall G, Berry L, Blisnick T, Braun BC (2008) The RhopH complex is transferred to the host cell cytoplasm following red blood cell invasion by *Plasmodium falciparum* . Mol Biochem Parasitol 160: 81–89. 18508137 10.1016/j.molbiopara.2008.04.002

[32] Desai SA (2012) Ion and nutrient uptake by malaria parasite-infected erythrocytes. Cell Microbiol 14: 1003–1009. 22432505 10.1111/j.1462-5822.2012.01790.x PMC3381363

[33] King LS, Yasui M, Agre P (2000) Aquaporins in health and disease. Mol Med Today 6: 60–65. 10652478 10.1016/s1357-4310(99)01636-6

[34] Kutner S, Breuer WV, Ginsburg H, Cabantchik ZI (1987) On the mode of action of phlorizin as an antimalarial agent in *in vitro* cultures of *Plasmodium falciparum* . Biochem Pharmacol 36: 123–129. 3099799 10.1016/0006-2952(87)90389-3

[35] Desai SA, Alkhalil A, Kang M, Ashfaq U, Nguyen ML (2005) PSAC-independent phloridzin resistance in *Plasmodium falciparum* . J Biol Chem 280: 16861–16867. 15701633 10.1074/jbc.M414629200

[36] Wagner MA, Andemariam B, Desai SA (2003) A two-compartment model of osmotic lysis in *Plasmodium falciparum*-infected erythrocytes. Biophys J 84: 116–123. 12524269 10.1016/S0006-3495(03)74836-X PMC1302597

[37] Lisk G, Pain M, Gluzman IY, Kambhampati S, Furuya T, et al (2008) Changes in the plasmodial surface anion channel reduce leupeptin uptake and can confer drug resistance in *P. falciparum*-infected erythrocytes. Antimicrob Agents Chemother 52: 2346–2354. 18443109 10.1128/AAC.00057-08 PMC2443925

[38] Pillai AD, Nguitragool W, Lyko B, Dolinta K, Butler MM, et al (2012) Solute restriction reveals an essential role for *clag3*-associated channels in malaria parasite nutrient acquisition. Mol Pharmacol 82: 1104–1114. 22949525 10.1124/mol.112.081224 PMC3502622

[39] Broman KW, Wu H, Sen S, Churchill GA (2003) R/qtl: QTL mapping in experimental crosses. Bioinformatics 19: 889–890. 12724300 10.1093/bioinformatics/btg112

[40] Lyko B, Hammershaimb EA, Nguitragool W, Wellems TE, Desai SA (2012) A high-throughput method to detect *Plasmodium falciparum* clones in limiting dilution microplates. Malar J 11: 124. 22531353 10.1186/1475-2875-11-124 PMC3352123

[41] Baumeister S, Winterberg M, Duranton C, Huber SM, Lang F, et al (2006) Evidence for the involvement of *Plasmodium falciparum* proteins in the formation of new permeability pathways in the erythrocyte membrane. Mol Microbiol 60: 493–504. 16573697 10.1111/j.1365-2958.2006.05112.x

[42] Alexandre JS, Xangsayarath P, Kaewthamasorn M, Yahata K, Sattabongkot J, et al (2012) Stable allele frequency distribution of the *Plasmodium falciparum clag* genes encoding components of the high molecular weight rhoptry protein complex. Trop Med Health 40: 71–77. 23264726 10.2149/tmh.2012-13 PMC3521051

[43] Narahashi Y, Shibuya K, Yanagita M (1968) Studies on proteolytic enzymes (pronase) of *Streptomyces griseus* K-1. II. Separation of exo- and endopeptidases of pronase. J Biochem 64: 427–437. 5707831 10.1093/oxfordjournals.jbchem.a128914

[44] Bullen HE, Crabb BS, Gilson PR (2012) Recent insights into the export of PEXEL/HTS-motif containing proteins in Plasmodium parasites. Curr Opin Microbiol 15: 699–704. 23092921 10.1016/j.mib.2012.09.008

[45] Li J, Waterhouse RM, Zdobnov EM (2011) A remarkably stable TipE gene cluster: evolution of insect Para sodium channel auxiliary subunits. BMC Evol Biol 11: 337. 22098672 10.1186/1471-2148-11-337 PMC3240667

[46] Sharma P, Wollenberg K, Sellers M, Zainabadi K, Galinsky K, et al (2013) An epigenetic antimalarial resistance mechanism involving parasite genes linked to nutrient uptake. J Biol Chem 288: 19429–19440. 23720749 10.1074/jbc.M113.468371 PMC3707646

[47] Mira-Martinez S, Rovira-Graells N, Crowley VM, Altenhofen LM, Llinas M, et al (2013) Epigenetic switches in *clag3* genes mediate blasticidin S resistance in malaria parasites. Cell Microbiol 15: 1913–1923. 23819786 10.1111/cmi.12162 PMC4621952

[48] Cortes A, Crowley VM, Vaquero A, Voss TS (2012) A view on the role of epigenetics in the biology of malaria parasites. PLoS Pathog 8: e1002943. 23271963 10.1371/journal.ppat.1002943 PMC3521673

[49] Fiedler F (1987) Effects of secondary interactions on the kinetics of peptide and peptide ester hydrolysis by tissue kallikrein and trypsin. Eur J Biochem 163: 303–312. 3643848 10.1111/j.1432-1033.1987.tb10801.x

[50] McConn J, Ku E, Himoe A, Brandt KG, Hess GP (1971) Investigations of the chymotrypsin-catalyzed hydrolysis of specific substrates. V. Determination of pre-steady state kinetic parameters for specific substrate esters by stopped flow techniques. J Biol Chem 246: 2918–2925. 5554299

[51] Staines HM, Alkhalil A, Allen RJ, De Jonge HR, Derbyshire E, et al (2007) Electrophysiological studies of malaria parasite-infected erythrocytes: current status. Int J Parasitol 37: 475–482. 17292372 10.1016/j.ijpara.2006.12.013 PMC2746352

[52] Iriko H, Kaneko O, Otsuki H, Tsuboi T, Su XZ, et al (2008) Diversity and evolution of the *rhoph1*/*clag* multigene family of *Plasmodium falciparum* . Mol Biochem Parasitol 158: 11–21. 18155305 10.1016/j.molbiopara.2007.11.004 PMC2268843

[53] Haerteis S, Krappitz M, Diakov A, Krappitz A, Rauh R, et al (2012) Plasmin and chymotrypsin have distinct preferences for channel activating cleavage sites in the gamma subunit of the human epithelial sodium channel. J Gen Physiol 140: 375–389. 22966015 10.1085/jgp.201110763 PMC3457690

[54] Armstrong CM, Bezanilla F, Rojas E (1973) Destruction of sodium conductance inactivation in squid axons perfused with pronase. J Gen Physiol 62: 375–391. 4755846 10.1085/jgp.62.4.375 PMC2226121

[55] Cohn JV, Alkhalil A, Wagner MA, Rajapandi T, Desai SA (2003) Extracellular lysines on the plasmodial surface anion channel involved in Na^+^ exclusion. Mol Biochem Parasitol 132: 27–34. 14563534 10.1016/j.molbiopara.2003.08.001

[56] Ocampo M, Rodriguez LE, Curtidor H, Puentes A, Vera R, et al (2005) Identifying *Plasmodium falciparum* cytoadherence-linked asexual protein 3 (CLAG 3) sequences that specifically bind to C32 cells and erythrocytes. Protein Sci 14: 504–513. 15659379 10.1110/ps.04883905 PMC2253410

