# Correction: Proteolysis at a Specific Extracellular Residue Implicates Integral Membrane CLAG3 in Malaria Parasite Nutrient Channels

**DOI:** 10.1371/journal.pone.0102283

**Published:** 2014-07-02

**Authors:** The PLOS ONE Staff

There is no affiliation indicated for the first author, Wang Nguitragool. Wang Nguitragool is affiliated with: Department of Molecular Tropical Medicine and Genetics, Faculty of Tropical Medicine, Mahidol University, Bangkok, Thailand. The publisher apologizes for this error, which was introduced during the typesetting process.
